# Effects of cold air dehydration on icefish water dynamics and macromolecular oxidation measured by low‐field nuclear magnetic resonance and magnetic resonance imaging

**DOI:** 10.1002/fsn3.2039

**Published:** 2020-12-03

**Authors:** Yingying Zhu, Li Zhang, Zhuyi Lin, Zhonghui Zhang, Yeting Cao, Hua Ru, Jun Yan, Shuxian Li, Zhong Li

**Affiliations:** ^1^ Department of Food Nutrition and Test Center of Food Nutrition and Safety Suzhou Vocational University Suzhou China; ^2^ Arg & Forestry Prod Deep Proc Technol & Equipment Co‐Innovation Center for the Sustainable Forestry in Southern China College of Forestry Nanjing Forestry University Nanjing China; ^3^ Suzhou Niumag Analytical Instrument Corporation Suzhou China

**Keywords:** cold air drying, dehydration, icefish, LF‐NMR, water dynamics

## Abstract

We have used low‐field nuclear magnetic resonance (LF‐NMR) and magnetic resonance imaging to measure water dynamics and migration, color, and texture profile (TPA) of icefish dried with hot and cold air methods. Relaxation time of T_21_, T_22,_ and T_23_, and the peak area of A_22_ and A_23_ decreased significantly during drying. The water signal intensity decreased from the surface to inner regions during drying. Color parameters of L* and b* values increased significantly, TPA parameters of hardness increased, cohesiveness decreased significantly, and moisture content decreased significantly during drying. We observed correlations between the moisture content, TPA, color, and NMR parameters. In addition, we found lower thiobarbituric acid reactive substances and carbonyl content of the dried icefish with cold air compared with hot air. The cold air drying method yielded better sensory quality, and LF‐NMR was a useful nondestructive method to determine the degree of drying and the quality of icefish.

## Graphical abstract

In this study, we found significant change of water status, color, TPA parameters, and moisture content of icefish samples during the drying process, and great correlations between the moisture content, TPA parameters, color parameters, and NMR parameters, suggesting that LF‐NMR could be as a rapid, effective, and nondestructive technique to assess the quality of icefish during different drying process.

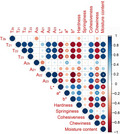

## 1 INTRODUCTION

Icefish of the Salangidae family exist primarily in the East China Sea, Bohai Sea, and their adjacent rivers or lakes in eastern Asia (Liu et al., 2018). China has the largest population of icefish (Zhang et al., 2013). Since 1979, the fish have been introduced from Taihu Lake in Suzhou City Jiangsu Province into other lakes and rivers throughout China (Kang et al., 2015). Icefish are one of the most popular Taihu Lake products available for aquaculture development. Aquaculture‐derived food is healthy because of its high protein and low fat content, and nutritional components such as vitamins, minerals, and extractable compounds (Cao et al., 2000). Fresh icefish is extremely perishable due to its high moisture content, abundant nutrients, and highly active enzymes. In China, 90% of all fresh icefish is processed into different kinds of products, including freeze‐dried and ready‐to‐eat items (Hu et al., 2010). Drying is a common process for the preservation and commercialization of icefish and related products.

Drying is an old topic in food processing. Drying can efficiently break the barrier of seasonal alternation and geographical variation, and it is a low cost and efficient method to preserve and prolong the shelf life of aquatic foods worldwide (Cheng et al., 2017; Tao et al., 2019). Because it does not require chemical preservatives, drying is also a safe and environmentally friendly production technology (Kilic, 2009). Sun drying and hot air drying are still the most common dehydration methods used by the food industry (Abhay et al., 2020).

Sun drying is affected mostly by weather conditions, contamination by microorganisms, dust, and insects, low drying efficiency, and high reduction in food nutritional value (Al‐Rubai et al., 2020; Selmi et al., 2010). A convective hot air dryer can provide temperatures from 40 to 90℃ for drying of different foods (Eminoglu et al., 2019). Higher temperature and a prolonged drying time cause biological and chemical reactions that degrade sensitive bioactive components. Other alterations occur, such as structural, mechanical, and physical modifications that affect color, sensory quality, nutrients, aroma, shape, and texture (Pokholchenko et al., 2020). Recently, cold air drying has been used to dry agricultural products. Cold air drying maintains the quality of fish with minimal fatty acid oxidation and protein denaturation and less nutrient degradation (Lewicki, 2006).

Drying is a complex process; temperature, time, and other factors affect the structural, sensorial, and nutritional attributes of products (Ben Haj Said et al., 2015). Water status, distribution, content, and its interactions influence the physical and chemical changes of materials during drying and storage (Younas et al., 2020). The investigation of water status can provide a road map of material during drying, which shows whether drying conditions are suitable. In many studies, only the overall water loss was measured instead of the changes in water status. During drying, it is difficult to monitor nondestructively the water status and distribution inside the materials.

Low‐field nuclear magnetic resonance (LF‐NMR) is a rapid and nondestructive method for visualization of water status and water distribution in food materials (Al‐Habsi et al., 2017; Li et al., 2015). The transverse relaxation time T_2_ provides the information on water status, water distribution, and the interaction between water and other macromolecules. Magnetic resonance imaging (MRI) provides the internal distribution of water and structural information in materials, and it shows the structural changes during drying (Xu et al., 2017). Yu et al. used LF‐NMR to investigate the water status and distribution in Chinese dried noodles during drying; the T_2_ of all water states followed a decreasing trend and the bound water was tightly combined with macromolecular materials that impeded water migration during drying (Xiaolei et al., 2018); Li et al. (2014) used LF‐NMR to study the water mobility of chicken breast during drying at different temperatures; the correlation between moisture content, the signal per mass, and the shear force value evaluated the drying degree and quality of chicken jerky. Tariq et al. used LF‐NMR and MRI to monitor the water dynamics and migration in Fuji apple slices during dehydration at various hot air oven temperatures; LF‐NMR exhibited great potential in evaluating the various water dynamics and quality of the apples during drying (Kamal et al., 2019). Zhang et al. used LF‐NMR to assess the feasibility of predicting the dielectric properties of Chinese yam slices; LF‐NMR was a rapid and noninvasive method, suitable for predicting dielectric properties (Li et al., 2019). Tan et al. used LF‐NMR and MRI to measure the dynamic states of water status and distribution in shrimp during drying; they observed correlations between moisture content, texture profile analysis (TPA), color, and LF‐NMR T_2_ parameters (Cheng et al., 2017). In sum, the LF‐NMR and MRI techniques are able to obtain much information about water mobility, water distribution, and structural and quality changes in food during drying.

In this study, we used LF‐NMR to analyze water mobility in icefish and illustrate the dynamic water states and distribution during hot air and cold air drying. In addition, we used MRI to obtain internal structural information of the icefish. We also monitored the moisture content, color, and TPA to assess the quality of dried icefish. Thiobarbituric acid reactive substances (TBARS) and carbonyl content were measured to assess lipid oxidation and protein oxidation, respectively, in the dried icefish. Correlations between LF‐NMR T_2_ relaxation parameters and the moisture content, color properties, and texture parameters (hardness, chewiness, cohesiveness, springiness) were performed to evaluate the potential of LF‐NMR as a nondestructive method to measure the drying degree and quality of icefish.

## 2 MATERIALS AND METHODS

### 2.1 Materials

Icefish about 5 ~ 6 cm and 2 ~ 3 g each were purchased from the local market in Xishan, Suzhou, China, transported to the laboratory in ice, and stored at −20℃. Before each experiment, the fish were thawed at 4°C for 24 hr and warmed to ambient air for 5 min for removing the water on the surface. The initial water content was 82.57 ± 4.32%, measured by 3 g minced icefish in a hot air dryer oven at 105°C until constant weight.

### 2.2 Dehydration process

After removing the surface water, each icefish was weighed and rapidly transferred for hot/cold air drying. The subsequent water content of the fish during drying was calculated by weight reduction. The icefish were divided into two groups, then dried at 10℃ (group C) and 40℃ (group H) with an air velocity of 0.2 m/s in a cold air dryer (Hangzhou Ouyi Electric Appliance Co., Ltd, Hangzhou, China) and a hot air drying oven (Shanghai Jinghong Experimental Equipment Co., Ltd). The number of replicates was 10 in both groups. NMR T_2_ relaxation of icefish was measured every hour during drying until the weight was reduced to 80%, to monitor the water dynamic changes. In addition, weight, color, and TPA were measured to monitor changes in quality. We used MRI to visualize the water distribution and internal structure of the icefish during drying. All measurements were performed in triplicate.

### 2.3 Water content analysis

The water in the hot‐ and cold‐dried icefish samples was measured by weighing every hour and calculated according to the following equation (1):

(1) $$W_{R}=\frac{W_{t}‐W_{e}}{W_{0}‐W_{e}}$$

where *W*_t_ is the immediate water content weight of icefish, *W*_0_ is the original water content, and *W*_e_ is the balanced water content of the dried icefish. The equation (1) can be simplified to the following equation (2):

(2) $$W_{R}=\frac{W_{t}}{W_{0}}$$

The value of *W*_e_ was assumed to be negligible compared with *W*_t_ and *W*_0_. The drying will stop when *W*_R_ is less than 20%.

### 2.4 LF‐NMR

Relaxation (T_2_) analysis was performed on a MesoMR23‐060H‐I NMR analyzer (Suzhou Niumag Analytical Instrument Co.) with a 0.5T permanent magnet and a 25 mm diameter radio frequency, corresponding to a proton resonance frequency of 23.40 MHz at 32 ± 0.01℃ with 90° and 180° pulses of 14 μs and 28 μs. Icefish were equilibrated to room temperature and placed in cylindrical glass tubes for transverse relaxation T_2_ measurements. Measurement conditions were the following: T_W_ (time waiting) = 3,000 ms, T_E_ (time echo) = 0.15 MS, NECH (number of echoes) = 5,000, NS (number of scan) = 8. Experimental temperature and humidity were 23.5℃ and 29.3%. We used NMR analysis software (Suzhou Niumag Analytical Instrument Co.) and Carr–Purcell–Meiboom–Gill (CPMG) pulse sequence to collect the relaxation signals. We used simultaneous iterative reconstruction 100,000 for inversion to obtain the relaxation map of the icefish samples.

### 2.5 MRI analysis

Magnetic resonance imaging was performed with the same LF‐NMR analyzer and a spin‐echo sequence. Icefish were placed in the center of the radio frequency coil, the signal was collected, and the T_2_‐weighted image was obtained. Image processing software was used to map and process the MRI image. The main parameters were the following: slice width = 4 mm, slice gap = 1 mm, T_R_ (time repetition) = 2,000, T_E_ (time echo) = 35 ms, average = 8, read size = 256, and phase size = 192.

### 2.6 Texture profile analysis

Texture profile analysis of icefish was tested with a Texture Analyzer (TA‐XT2i, British Stable Micro System Ltd.) fitted with a cylindrical probe (P/2). For TPA, the belly of the icefish was cut from samples dehydrated for different times. The parameters were the following: pretest speed of 0.2 mm/s, post‐test speed of 0.2 mm/s, test speed of 0.2 mm/s, trigger force of 5.0 g, 2 cycles of compression analysis, compression level of 30%, and relaxation time of 5 s between the two cycles. The probe was recovered to the trigger point before the second cycle and returned to the initial position after the second cycle. The tests were performed in triplicate. TPA data were analyzed automatically by the texture expert software (British Stable Micro System Ltd.). The typical texture variables of icefish samples that we measured were hardness, springiness, chewiness, and cohesiveness.

### 2.7 Color measurement

The color parameter of icefish was measured by color analyzer (CR‐200, Konica Minolta Investment Ltd.). Icefish samples were equilibrated to ambient temperature before color measurements. The belly section of the fish was used for color measurements. The color parameters of L^*^, a^*^, and b^*^ were used to evaluate the difference in color change. L^*^ represents lightness from black to white, a^*^ is from green to red, and b^*^ is from blue to yellow. All experiments were performed in triplicate.

### 2.8 Thiobarbituric acid reactive substances

Thiobarbituric acid reactive substances are an index of lipid oxidation. TBARS was determined after drying (Sun et al., 2001). Dried icefish sample (0.5 g) was added to 10 ml 10% (w/v) trichloroacetic acid (containing 0.1% tetra‐ethylamine acetate) and homogenized for 30 s with a high‐speed tissue homogenizer (10,000 rev/min). The homogenate was filtered, and 3 ml of filtrate was mixed with 3 ml of 0.02 M thiobarbituric acid in a water bath at 100 ℃ for 45 min. After cooling to room temperature, the absorbance of the sample was measured at 532 nm; 1,1,3,3‐tetraethoxypropane was used as the absorbance standard. All experiments were performed in triplicate. The results were expressed as mg malondialdehyde (MDA)/kg dried icefish.

### 2.9 Protein carbonyl content

Protein carbonyl content is a measure of protein oxidation (Xiong et al., 2000). Protein carbonyl content in dried icefish samples was determined as follows (Mercier et al., 1998): Dried icefish (0.5 g) was mixed with 10 ml 2% SDS, 0.01 M sodium phosphate, pH 7.0. The samples were homogenized and clarified by centrifugation at 3,000 g. Protein concentration of the supernatant was determined at 278 nm with BCA in guanidine as standard. Two mL of 2 M HCl containing 10 mM 2,4‐dinitrophenylhydrazine was added to the protein extract, which was then heated for 1 hr at 30 ℃, stirring every 10 min. Then, 10% (w/v) trichloroacetic acid was added, the sample was centrifuged at 11,000 *g* for 15 min, and the supernatant was discarded. The pellet was washed twice with ethanol:ethyl acetate (v/v = 1:l) to remove unreacted dinitrophenylhydrazine. The pellet was dissolved in 3 ml 6 M guanidine HCl (pH 6.5, with 20 mM sodium phosphate buffer), and the absorbance of the dissolved sample was measured at 370 nm (representing carbonyl substances). The carbonyl content was calculated from the carbonyl molar extinction coefficient of 22,000 m^‐1^/cm. The results were expressed as nmol carbonyl/mg protein.

### 2.10 Statistical analysis

The data were analyzed by SAS 9.2, and the analysis of variance was adopted by one‐way ANOVA and Duncan's multiple comparisons. The results were expressed as mean ± standard deviation. The correlations of NMR T_2_ relaxation parameters, color properties, texture profile, and moisture content were analyzed by R 3.6.3 using Hmisc and Corrplot packages.

## 3 RESULTS AND DISCUSSION

### 3.1 Moisture content of icefish during drying

**Figure 1:**
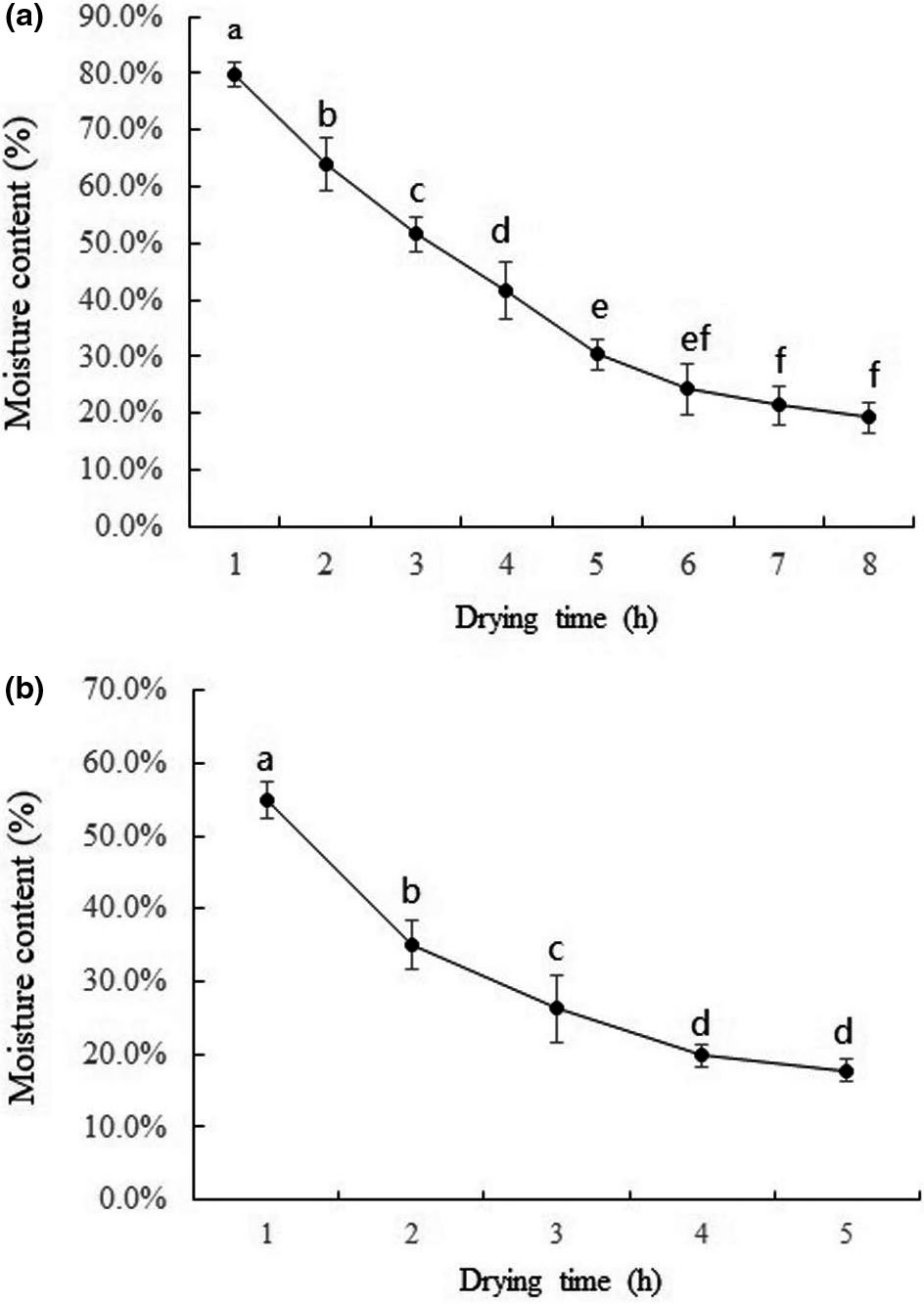
Changes of moisture content of icefish during drying process. (a) Group C. (b) Group H. *Note:* Standard error bars are indicated, and different letters on the bars represent significant differences (*p* < .05) in each treatment (*n* = 10)

Figure 1 shows the moisture content of icefish during drying. Moisture content was reduced in a time‐dependent manner, whether we used hot air or cold air drying. For cold air drying at 8°C, the moisture content at 1, 2, 3, 4, 5, 6, 7, and 8 hr was, respectively, 79.68 ± 2.09%, 63.92 ± 4.81%, 51.60 ± 3.03%, 42.62 ± 5.02%, 36.40 ± 2.66%, 29.26 ± 4.46%, 23.32 ± 3.35%, and 19.18 ± 2.57%. For hot air drying at 40℃, the values for moisture content at 1, 2, 3, 4, and 5 hr were 55.01 ± 2.6%, 35.1 ± 3.4%, 27.3 ± 4.6%, 21.8 ± 1.4%, and 18.8 ± 1.4%. As expected, the drying rate of icefish with hot air was faster than that with cold air due to a greater evaporation rate.

### 3.2 Water status and distribution at different drying

Water removal from icefish could affect water status and distribution during drying. Low‐field NMR and MRI are optimum, nondestructive methods to identify the degree of change in water status and microstructure. The water status, content, and distribution are the most critical factors that affect the quality of dehydrated foods. The T_2_ relaxation spectra can be used to measure those three parameters (Ezeanaka et al., 2019).

**Figure 2:**
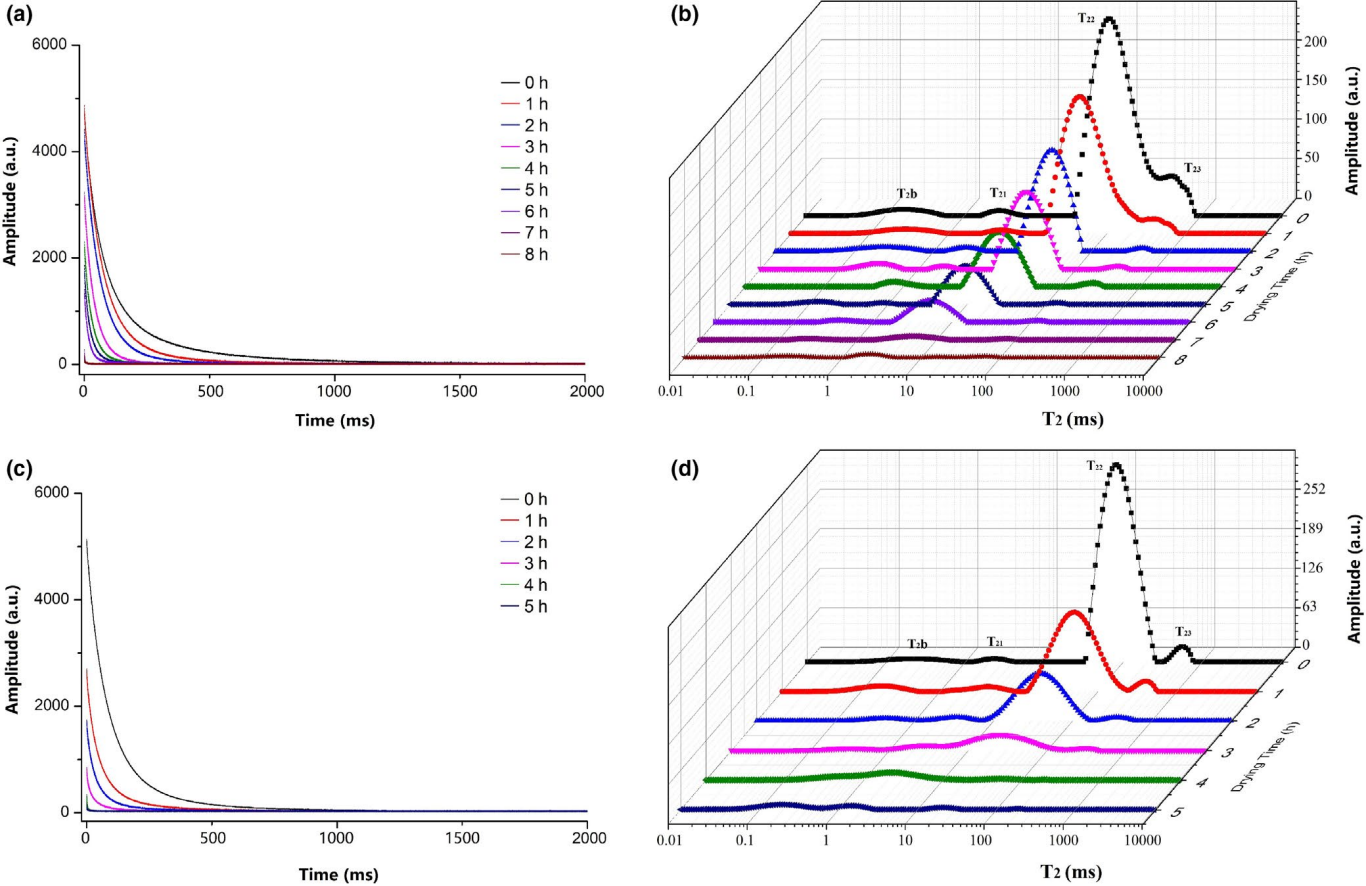
Carr–Purcell–Meiboom–Gill (CPMG) signal and T_2_ relaxation spectra of icefish during drying process. (a) CPMG signal of icefish changes during cold air drying process. (b) CPMG signal of icefish changes during hot air drying process. (c) T_2_ relaxation spectra of icefish during cold air drying process. (d) T_2_ relaxation spectra of icefish during hot air drying process

Figure 2a,b shows the Carr–Purcell–Meiboom–Gill (CPMG) pulse sequence decay curves of icefish for different hot and cold air drying times. The CPMG signals decayed faster with the extension of drying time during 1 hr in group H. After 1 hr, the drying rate decreased slightly because of little water content remaining in the samples. When the icefish samples were dried with cold air, the CPMG signals decayed faster with the extension of drying time during 1 ~ 3 hr and then decay slowed. Compared with hot air drying, the decreased rate of loss of moisture content in icefish was slower in group C, which was consistent with the decrease of moisture content in icefish. The results showed greatly decreased water mobility during drying, whether with hot air or cold air drying. The internal moisture content was high in fresh icefish samples (84.03 ± 2.31%), and it decreased with the extension of hot air or cold air drying time, which diminished the NMR signal and gradually accelerated attenuation rate.

The T_2_ relaxation spectra of icefish during cold air drying (Figure 2c) and hot air drying (Figure 2d) showed four peaks, T_2b_, T_21_, T_22,_ and T_23_. There were three different water statuses of icefish during drying: bound water T_2b_ and T_21_, immobilized water entrapped within the myofibril lattice T_23_, and free water T_24_ in the myofibril lattice (Cheng et al., 2017; Tan et al., 2018; Wang et al., 2018; Xu et al., 2017). Relaxation T_2b_ corresponded to water strongly bound to polar groups of icefish protein molecules. Relaxation T_21_ was lightly bound water trapped within highly organized structures (Cheng et al., 2017). With the extension of drying time, the relaxation time of T_21_, T_22,_ and T_23_ decreased gradually for both hot and cold air drying. The relaxation time of T_2_ decreased, which implied that water had a low degree of freedom of movement (Shao & Li, 2012).

To understand comprehensively the dynamic changes and migration of different water phases, we calculated the quantitative data of relaxation time of T_2b_, T_21_, T_22_, and T_23_ and values of peak area of each fraction A_2b_, A_21_, A_22_, and A_23_ (see Table 1). We found a small leftward shift of T_2b_ and T_21_ during drying, which indicated that water mobility was influenced somewhat by the extension of drying time. Relaxation T_2b_ did not change significantly (*p* > .05) in the two groups. This result showed that the water mobility remained unchanged during the entire air drying process. We observed a significant decrease in T_22_ in groups C and H, which suggested that the water mobility or freedom of immobilized water was reduced gradually during drying. It was interesting to find that T_21_ disappeared in the later stage of drying, at 4 and 7 hr in group H and group C. This disappearance was probably because drying accelerated the left shift of T_22_, which caused it to overlap and obscure T_21_. Due to the high mobility and weak intensity of immobilized water, relaxation T_23_ decreased significantly and disappeared after 4 hr in group H and 7 hr in group C. There was no regular change (rising or falling) for A_2b_ in group H, whereas A_2b_ decreased slightly, then increased, in group C during drying. This result indicated that the strongly bound water was influenced by the cold air drying. A_22_ decreased significantly in group H and group C with the extension of the drying time, which indicated a significant decrease in immobilized water during drying. A_23_ decreased significantly, then disappeared at 4 hr in group H and 7h in group C, which indicated a decrease in immobilized water. The mobility and freedom of water in icefish had converted from a high level to a low level, and significant moisture loss was observed from the reduction in A_22_.

**Table 1:** LF‐NMR parameters of icefish at different drying times

Group	Drying time (hr)	T_2b_ (ms)	T_21_ (ms)	T_22_ (ms)	T_23_ (ms)	A_2b_	A_21_	A_22_	A_23_
C	0	0.40 ± 0.09^a^	5.76 ± 1.23^a^	58.75 ± 6.21^a^	540.26 ± 46.69^a^	42.39 ± 13.72^a^	28.19 ± 3.07^a^	2,190.67 ± 97.04^a^	126.37 ± 32.51^a^
	1	0.41 ± 0.07^a^	4.88 ± 0.03^a^	44.49 ± 3.26^ab^	363.13 ± 16.22^b^	39.78 ± 10.92^ab^	33.17 ± 6.55^a^	1,406.13 ± 47.26^b^	69.36 ± 27.04^b^
	2	0.36 ± 0.12^a^	4.03 ± 0.09^ab^	32.18 ± 5.43^b^	285.63 ± 22.09^b^	31.08 ± 8.62^bc^	39.43 ± 10.03^a^	930.01 ± 22.17^c^	30.75 ± 15.49^c^
	3	0.32 ± 0.15^a^	3.49 ± 1.01^b^	28.97 ± 2.27^b^	252.16 ± 18.16^c^	25.07 ± 13.82^c^	27.55 ± 8.53^a^	572.55 ± 19.08^d^	23.71 ± 8.56^c^
	4	0.39 ± 0.33^a^	3.01 ± 0.08^b^	23.82 ± 6.18^b^	219.92 ± 17.46^c^	33.35 ± 6.59^b^	19.86 ± 6.92^b^	375.91 ± 33.23^e^	16.53 ± 3.03^cd^
	5	0.35 ± 0.19^a^	2.57 ± 0.29^b^	17.73 ± 2.03^c^	165.38 ± 9.01^cd^	38.23 ± 7.59^ab^	15.27 ± 3.05^bc^	253.86 ± 17.70^e^	13.79 ± 6.69^d^
	6	0.29 ± 0.04^a^	2.21 ± 0.79^b^	13.11 ± 0.79^c^	115.21 ± 30.07^d^	40.36 ± 5.02^ab^	10.35 ± 5.06^c^	189.41 ± 6.79^e^	9.52 ± 3.03^d^
	7	0.25 ± 0.10^a^	—	10.62 ± 1.11^c^	—	46.16 ± 7.60^a^	—	117.80 ± 37.62^f^	—
	8	0.21 ± 0.09^a^	—	8.01 ± 1.03^c^	—	54.98 ± 13.83^a^	—	95.22 ± 31.07^f^	—
H	0	0.43 ± 0.16^a^	5.17 ± 3.21^a^	61.11 ± 5.27^a^	580.92 ± 62.18^a^	47.95 ± 14.76^a^	29.89 ± 3.59^b^	2,219.23 ± 109.31^a^	117.16 ± 28.12^a^
	1	0.40 ± 0.21^a^	4.20 ± 1.79^a^	51.11 ± 4.92^ab^	410.26 ± 15.03^b^	32.36 ± 13.62^b^	47.21 ± 8.32^a^	766.01 ± 35.20^b^	57.53 ± 13.10^b^
	2	0.30 ± 0.11^a^	3.65 ± 0.96^ab^	38.72 ± 6.09^b^	301.26 ± 12.31^c^	33.16 ± 12.82^b^	59.62 ± 9.75^a^	346.22 ± 43.55^c^	27.11 ± 3.26^c^
	3	0.27 ± 0.09^a^	2.77 ± 1.34^b^	22.15 ± 4.61^c^	232.01 ± 29.52^c^	45.37 ± 21.22^a^	26.13 ± 8.53^b^	157.83 ± 18.92^cd^	10.09 ± 1.22^c^
	4	0.29 ± 0.12^a^	—	15.53 ± 2.28^cd^	—	53.82 ± 19.32^a^	—	121.51 ± 23.33^d^	—
	5	0.37 ± 0.17^a^	—	8.67 ± 1.02^d^	—	43.98 ± 13.83^ab^	—	85.3 ± 39.67^d^	—

Note

Different letters of superscripts in each column represent significant differences (*p* < .05) in each treatment (*n* = 10).

### 3.3 MRI analysis of icefish during the drying

**Figure 3:**
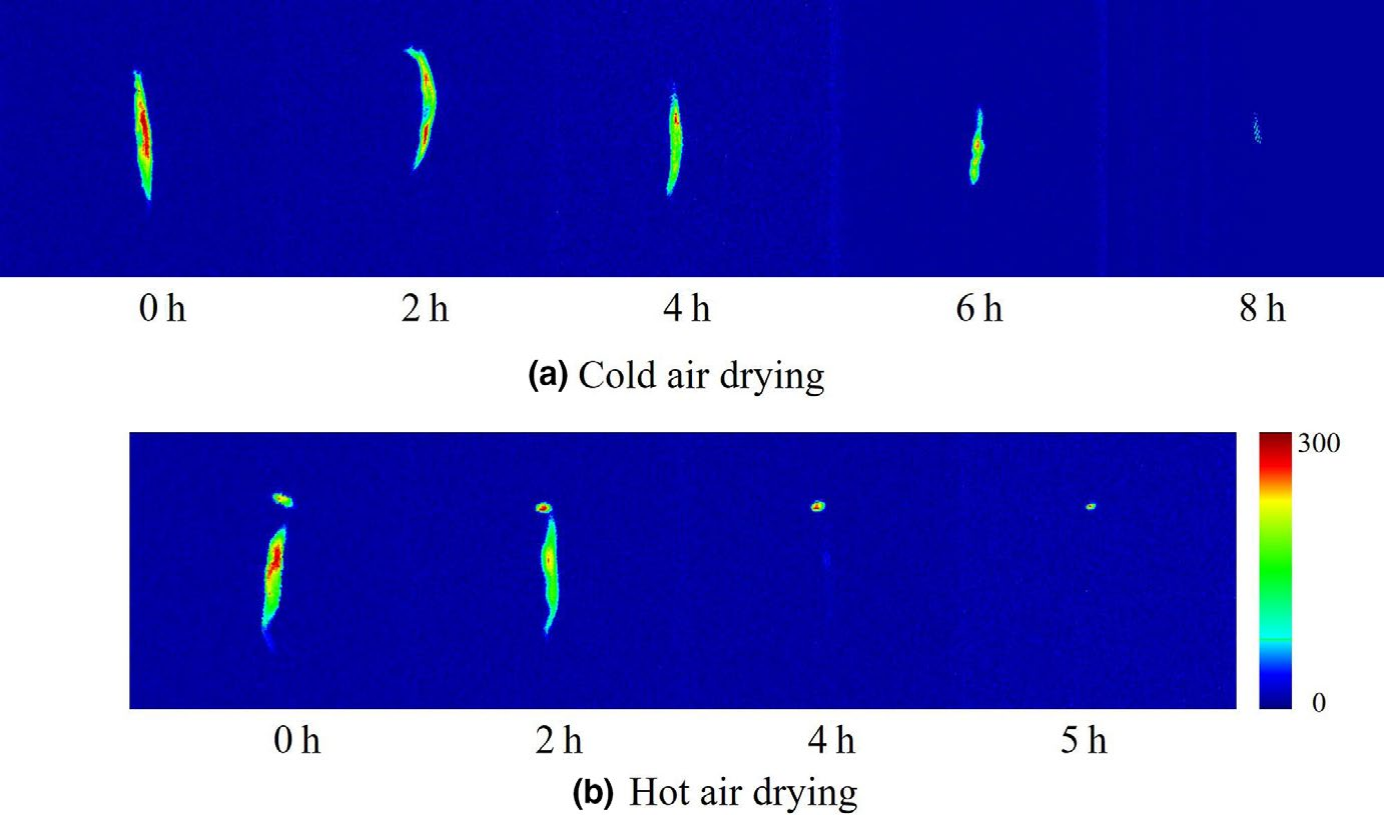
T_2_‐weighted MRI images of dried icefish of different drying process. (a) MRI images of icefish samples during cold air drying process. (b) MRI images of icefish samples during hot air drying process

Magnetic resonance imaging can visualize the water distribution and internal structure in food samples and display changes intuitively during various food processing methods (Fan et al., 2017; Li et al., 2018; Xu et al., 2017). According to different signal strengths of different points on the cross section of the samples, a pixel with a large signal has greater brightness and a smaller signal has smaller brightness; these pixels are combined and pseudocolor‐processed to obtain the MRI. Red represents a high density of protons and blue represents a low density of protons in the corresponding curves related to the intensities of T_2_‐weighted images. The changes in water distribution and internal structural information in icefish samples at 0, 2, 4, 6, and 8 hr in group H and 0, 2, 4, and 5 hr in group C are shown in Figure 3a,b as pseudocolor images. The red color of MRI in all icefish samples was reduced toward blue with the extension of drying time. The T_2_‐weighted images of icefish at 6 hr in group C showed that there was still some water in the belly of the fish, and it nearly disappeared at 8 hr. The loss of MRI signals in group H was faster than in group C, and only parts of signals of the head of icefish samples were found at 4 hr in group C. The icefish samples in group H and group C were almost completely dried at 5 hr and 8 hr, respectively. In cold air, icefish samples required more time for removing water. The decreased moisture content of icefish was directly related to the drying method, drying time, and the signal intensities of the T_2_‐weighted images in accordance with a significant water distribution changes of icefish in drying. MRI provides visualization of the drying status of icefish during drying.

### 3.4 Color of icefish during drying

**Figure 4:**
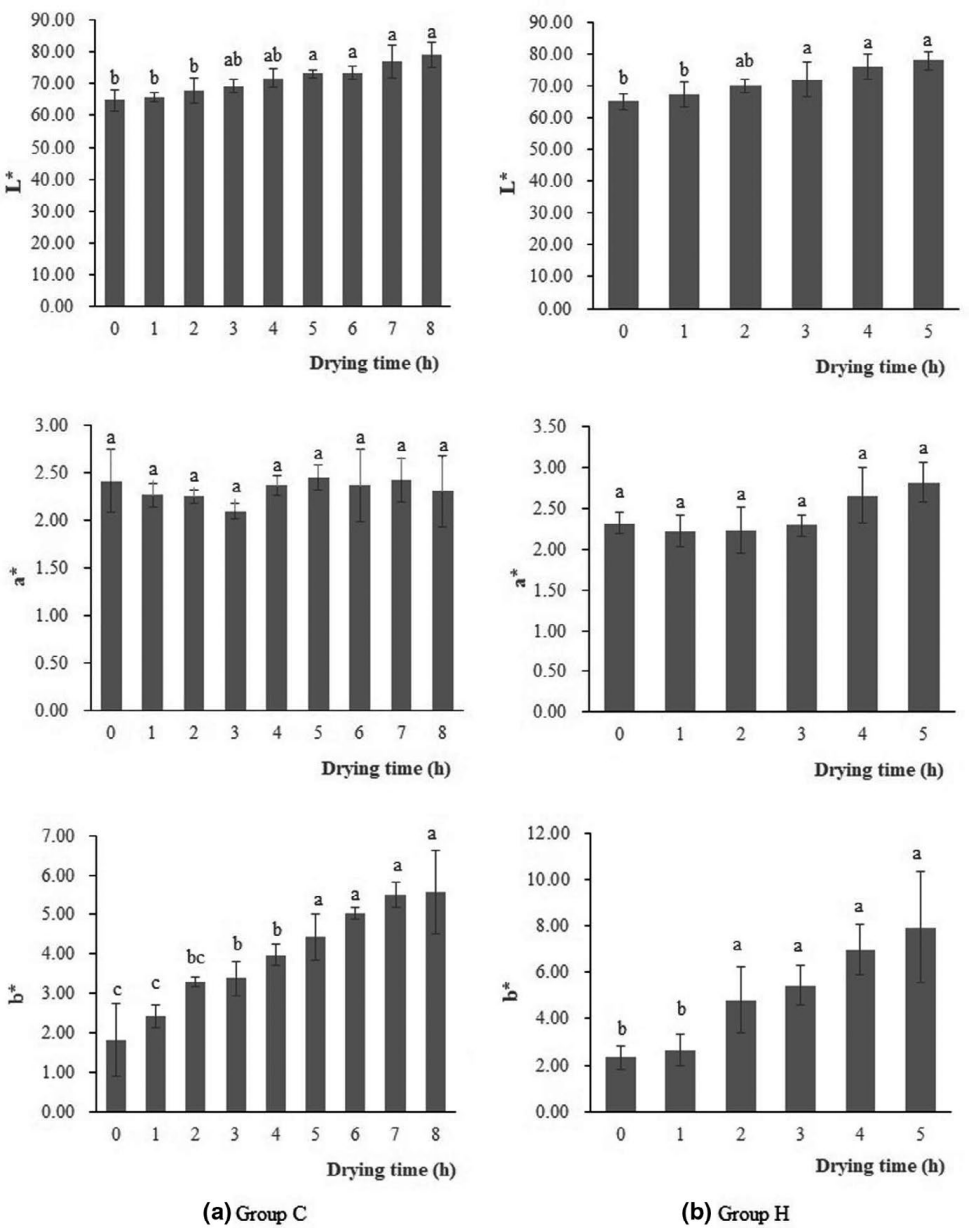
Changes of color parameters of icefish. (a) Changes of color parameters of icefish in group C. (b) Changes of color parameters of icefish in group. *Note:* Standard error bars are indicated, and different letters on the bars represent significant differences (*p* < .05) in each treatment (*n* = 10)

Color is one of the main contributors of dried foodstuff sensory quality and acceptability. L^*^ represents the brightness intensity of white and exhibits the degree of lightness, a^*^ represents redness intensity and exhibits the degree of redness, and b^*^ represents yellowness intensity and exhibits the degree of yellowness. Figure 4 shows the results of L^*^, a^*^, and b^*^ for the icefish of group C and group H. The color parameters varied during drying. The L^*^ and b^*^ values increased significantly (*p* < .05) with the extension of drying time, which suggested that icefish became lighter and more yellow at the end of drying. The reason for lightening was that a fresh icefish is transparent and becomes white as drying proceeds. For a^*^ values, we did not observe any significant changes during drying. The color measurements indicated that group C had a low value of b^*^and a high value level of L^*^, which demonstrated that the cold air‐dried icefish had better sensory quality.

### 3.5 TPA of icefish during drying

Texture, particularly hardness, is one of the most prominent parameters that affects dried food sensory quality. Table 2 shows the TPA parameters for hardness, springiness, cohesiveness, and chewiness of icefish during drying. Hardness and chewiness increased significantly with the extension of drying time in both groups, especially during the final period of drying. The hardness value of 79.76 ± 12.56 increased to 3,679.32 ± 115.48 in group C and from 82.12 ± 9.27 to 4,832.21 ± 107.10 in group H, that is, increases by factors of 46 and 59, respectively, during cold and hot air drying. The principal reason for increased hardness was probably the substantial removal of water during drying that induced structural changes in icefish cells or tissue, creating a compact microstructure and greater hardness and chewiness. The TPA parameters of cohesiveness decreased gradually in the two groups during drying. The dried icefish samples were more fragile than fresh samples, which may explain the decreased cohesiveness. The two groups did not differ in springiness.

**Table 2:** Changes of TPA parameters of icefish

Group	Drying time (hr)	Hardness	Springiness	Cohesiveness	Chewiness
C	0	79.76 ± 12.56^d^	0.84 ± 0.03^a^	0.84 ± 0.02^a^	38.182 ± 11.22^e^
	1	105.62 ± 18.76^d^	0.85 ± 0.02^a^	0.81 ± 0.04^a^	52.71 ± 16.87^e^
	2	181.63 ± 17.95^cd^	0.83 ± 0.03^a^	0.79 ± 0.02^a^	96.53 ± 22.98^e^
	3	258.95 ± 32.66^c^	0.82 ± 0.01^a^	0.76 ± 0.02^a^	173.30 ± 13.88^de^
	4	586.49 ± 53.2^c^	0.81 ± 0.02^a^	0.73 ± 0.02^b^	265.75±±27.85^d^
	5	789.63 ± 67.51^c^	0.81 ± 0.05^a^	0.72 ± 0.02^b^	516.76 ± 19.92^cd^
	6	1,279.32 ± 58.66^c^	0.80 ± 0.02^a^	0.71 ± 0.03^b^	1,000.12 ± 36.23^c^
	7	1,959.03 ± 74.82^b^	0.79 ± 0.03^a^	0.69 ± 0.04^b^	1,331.49 ± 87.16^b^
	8	3,679.32 ± 115.48^a^	0.80 ± 0.03^a^	0.67 ± 0.03^b^	1,867.32 ± 152.07^a^
H	0	82.12 ± 9.27^e^	0.86 ± 0.03^a^	0.84 ± 0.28^a^	41.81 ± 4.76^e^
	1	201.89 ± 22.91^e^	0.85 ± 0.02^a^	0.78 ± 003^b^	63.72 ± 5.27^e^
	2	594.50 ± 50.5^d^	0.83 ± 0.02^a^	0.76 ± 0.04^b^	108.63 ± 27.89^d^
	3	970.42 ± 53.36^c^	0.82 ± 0.09^a^	0.71 ± 0.05^c^	435.95 ± 30.24^c^
	4	2,239.03 ± 74.21^b^	0.80 ± 0.04^b^	0.70 ± 0.03^c^	1,001.13 ± 38.69^b^
	5	4,832.21 ± 107.10^a^	0.79 ± 0.04^b^	0.66 ± 0.08^c^	1,533.44 ± 99.15^a^

Note

Different letters of superscripts in each column represent significant differences (*p* < .05) in each treatment (*n* = 10).

### 3.6 Effects of different drying methods on icefish lipid and protein oxidation

**Figure 5:**
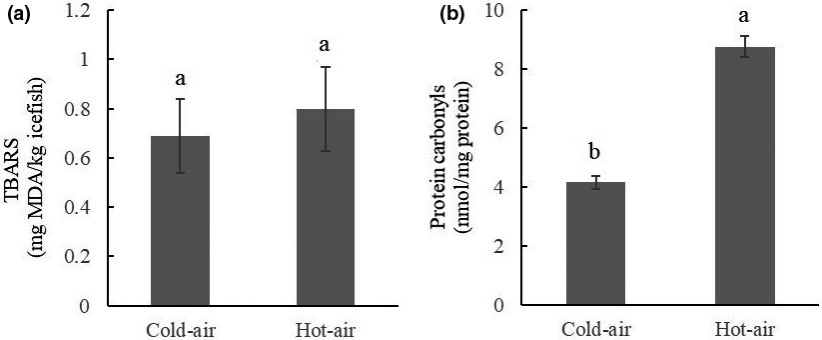
Oxidation indexes of dried icefish in different drying processes. (a) TBARS value. (b) Protein carbonyl content. *Note:* Standard error bars are indicated, and different letters on the bars represent significant differences (*p* < .05) in each treatment (*n* = 10)

Lipid oxidation is a free radical chain reaction. The primary oxidation products are conjugated dienes and hydroperoxides, which degrade easily into secondary oxidation products such as ketones, alcohols, and aldehydes. Thiobarbituric acid reactive substances are the most common method for determination of lipid secondary oxidation products such as malondialdehyde (MDA). Carbonylation is a significant characteristic of protein oxidation. Figure 5 shows results of TBARS and protein carbonyl measurements. The TBARS value was 0.69 ± 0.17 mg MDA/kg in group C and 0.80 ± 0.18 mg MDA/kg in group H. The protein carbonyl content was 0.69 ± 0.17 nmol/mg protein in group C and 0.80 ± 0.18 nmol/mg protein in group H. The two groups did not differ in TBARS value (*p* > .05), but they differed in protein carbonyl content (*p* < .05). Compared with hot air drying, cold air drying significantly reduced oxidation of fat and protein in dried icefish, and it improved the quality of the dried fish.

### 3.7 Correlation analysis of TPA parameters, color parameters, and NMR data

**Figure 6:**
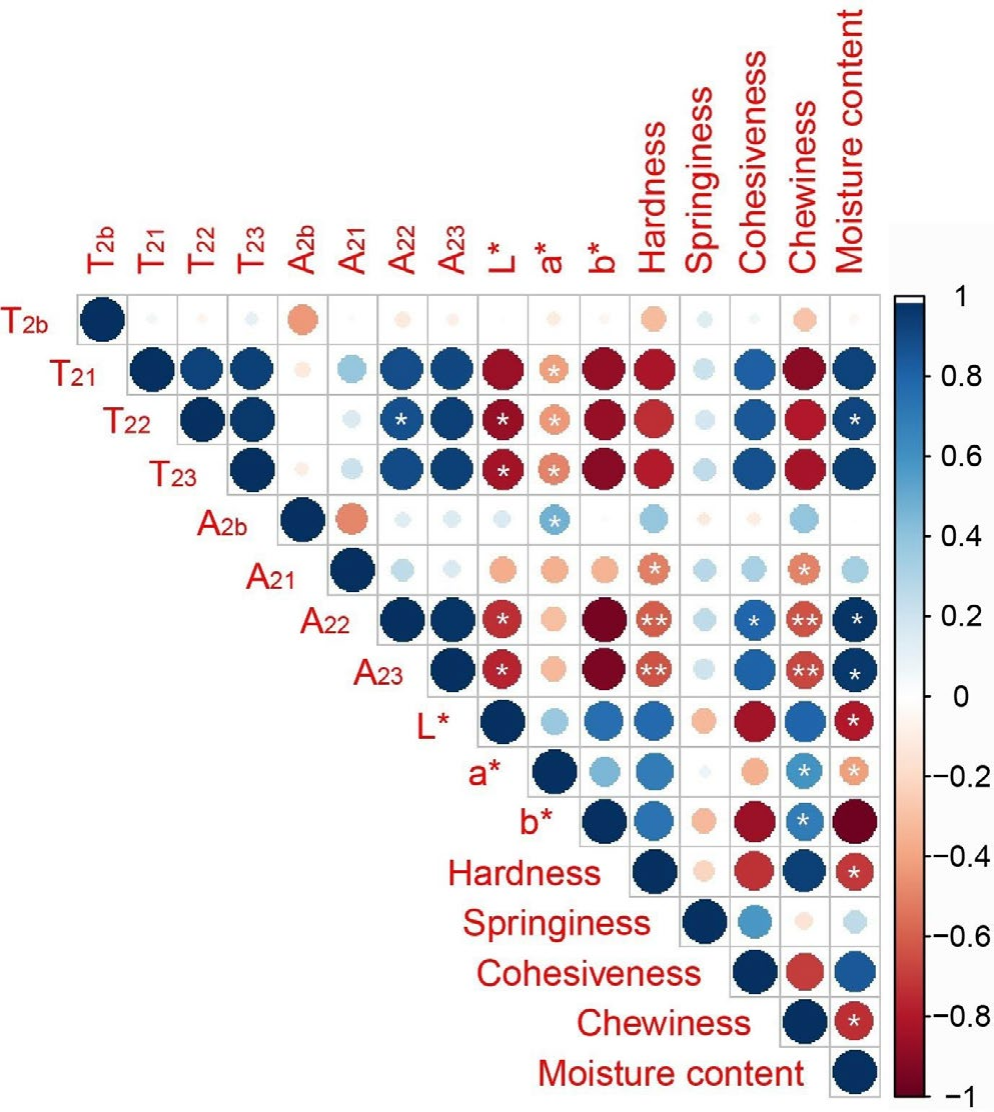
Correlations of TPA parameters, color parameters, and NMR data. *Note:* * in circles represents significant differences (*p* < .05), and ** in circles represents significant differences (*p* < .01)

To identify relationships between color, TPA parameters, and NMR data, we used R 3.6.3 to analyze the four TPA parameters (hardness, springiness, cohesiveness, and chewiness), the three color parameters (L^*^, a^*^, and b^*^), and the eight LF‐NMR T_2_ parameters (T_2b_, T_21_, T_22_, T_23_, A_2b_, A_21_, A_22,_ and A_23_) (Figure 6). L^*^ and b^*^ were negatively correlated with LF‐NMR T_2_ parameters (T_22_, T_23_, A_22_, and A_23_), and a^*^ was positively correlated with LF‐NMR T_2_ parameters (A_2b_). Hardness and chewiness were negatively correlated with LF‐NMR T_2_ parameters (T_22_, T_23_, A_22,_ and A_23_), whereas cohesiveness was positively correlated with LF‐NMR T_2_ parameters. The significant correlations between LF‐NMR T_2_ parameters (especially of A_22_ and A_23_) and color/TPA parameters indicated that LF‐NMR was a reliable technique to monitor changes of color and TPA parameters for icefish during the drying.

## 4 CONCLUSION

We measured the moisture content, color, and TPA parameters of icefish subjected to two drying methods. In addition, we used LF‐NMR and MRI to assess changes in water mobility and distribution in the fish. Drying of icefish had significant effects on moisture content, water mobility, water freedom, color, and TPA parameters with extension of the drying time. The significant decrease of T_2_ relaxation time indicated that water mobility was reduced because of loss of water content during drying. As drying proceeded, the signal per mass of immobilized water (A_22_) and bulk water (A_23_) decreased significantly, which indicated that water content gradually diminished, consistent with the results of water loss. MRI results provided water distributions and internal structure information of icefish during drying; the water signal intensity decreased from the surface to inner regions. In addition, the cold air drying method significantly reduced the oxidation of fat and protein in dried icefish and improved the quality. Thus, a cold air dryer could be used in the commercial production of dried icefish. In addition, significant correlations were observed between the moisture content, TPA parameters, color parameters, and NMR parameters, which suggested that LF‐NMR is a rapid, effective, and nondestructive technique to assess water content, color, and TPA in dried icefish.

## CONFLICT OF INTEREST

There was no competing interests in this manuscript.

## Data Availability

The data that support the findings of this study are available from the corresponding author upon reasonable request.
